# 3D cephalometric analysis using Magnetic Resonance Imaging: validation of accuracy and reproducibility

**DOI:** 10.1038/s41598-018-31384-8

**Published:** 2018-08-29

**Authors:** Alexander Juerchott, Muhammad Abdullah Saleem, Tim Hilgenfeld, Christian Freudlsperger, Sebastian Zingler, Christopher J. Lux, Martin Bendszus, Sabine Heiland

**Affiliations:** 1 Department of Neuroradiology, Heidelberg University Hospital, Im Neuenheimer Feld 400, 69120 Heidelberg, Germany; 2 Division of Experimental Radiology, Department of Neuroradiology, Heidelberg University Hospital, Im Neuenheimer Feld 400, 69120 Heidelberg, Germany; 3 Department of Oral and Maxillofacial Surgery, Heidelberg University Hospital, Im Neuenheimer Feld 400, 69120 Heidelberg, Germany; 4 Department of Orthodontics and Dentofacial Orthopedics, Heidelberg University Hospital, Im Neuenheimer Feld 400, 69120 Heidelberg, Germany

## Abstract

The aim of this study was to validate geometric accuracy and *in vivo* reproducibility of landmark-based cephalometric measurements using high-resolution 3D Magnetic Resonance Imaging (MRI) at 3 Tesla. For accuracy validation, 96 angular and 96 linear measurements were taken on a phantom in 3 different positions. *In vivo* MRI scans were performed on 3 volunteers in five head positions. For each *in vivo* scan, 27 landmarks were determined from which 19 angles and 26 distances were calculated. Statistical analysis was performed using Bland-Altman analysis, the two one-sided tests procedure and repeated measures one-way analysis of variance. In comparison to ground truth, all MRI-based phantom measurements showed statistical equivalence (p < 0.001) and an excellent agreement in Bland-Altman analysis (bias ranges: −0.090–0.044°, −0.220–0.241 mm). *In vivo* cephalometric analysis was highly reproducible among the five different head positions in all study participants, without statistical differences for all angles and distances (p > 0.05). Ranges between maximum and minimum *in vivo* values were consistently smaller than 2° and 2 mm, respectively (average ranges: 0.88°/0.87 mm). In conclusion, this study demonstrates that accurate and reproducible 3D cephalometric analysis can be performed without exposure to ionizing radiation using MRI.

## Introduction

Introduced in the early 1930s, cephalometric analyses based on lateral and frontal radiographs have remained basic elements of routine diagnostics in orthodontics and orthognathic surgery. However, the diagnostic benefit of 2D cephalometric radiographs in diagnosis and treatment planning is limited due to geometric distortions, superimpositions and the dependence on correct head positioning^1–4^. In contrast, cone-beam computed tomography (CBCT) enables craniofacial measurements in three dimensions with high geometric accuracy^5–7^. Along with the rapid technical development and the continuously increasing availability, CBCT has also moved into focus for 3D cephalometric analysis^8–10^. However, radiation exposure caused by CBCT is substantially higher compared to conventional radiographs^11^ and therefore indications are restricted to severe clinical cases^10^. This constraint also results in limited evidence for diagnostic efficacy of 3D cephalometric analysis so far^8,10^.

Even though Magnetic Resonance Imaging (MRI) has become one of the most important modalities in medical imaging and is routinely used in the head and neck region, it has not been established as a tool for 3D treatment planning in orthodontics and orthognathic surgery so far. Recently, however, considerable progress has been made in the application of MRI in craniofacial imaging through the development of high-field scanners, dedicated coil systems^12,13^ and application-optimized sequences^14,15^. In particular, novel 3D sequences with high spatial resolution make MRI a promising modality for cephalometric analysis^16^, and *in vitro* studies have already been able to demonstrate that MRI allows for accurate osteometric measurements in the maxillofacial area^17–21^. Thus, MRI may resolve the dilemma between the limited diagnostic opportunities of conventional radiographs and the high radiation exposure of CBCT. However, to the best of our knowledge, the role of MRI in 3D cephalometric analysis has not yet been investigated. To address this, we developed a time-efficient, high-resolution MRI technique and a dedicated software tool. Using this methodology, the purpose of the present study was to validate geometric accuracy and reproducibility of MRI in landmark-based 3D cephalometric analysis in two consecutive steps: First, the accuracy of angular and linear measurements was analyzed *in vitro* by scanning a cuboid phantom in different positions and comparing results to ground truth. Second, the reproducibility of 3D cephalometric measurements in various head positions was evaluated *in vivo*.

## Results

**Figure 1:**
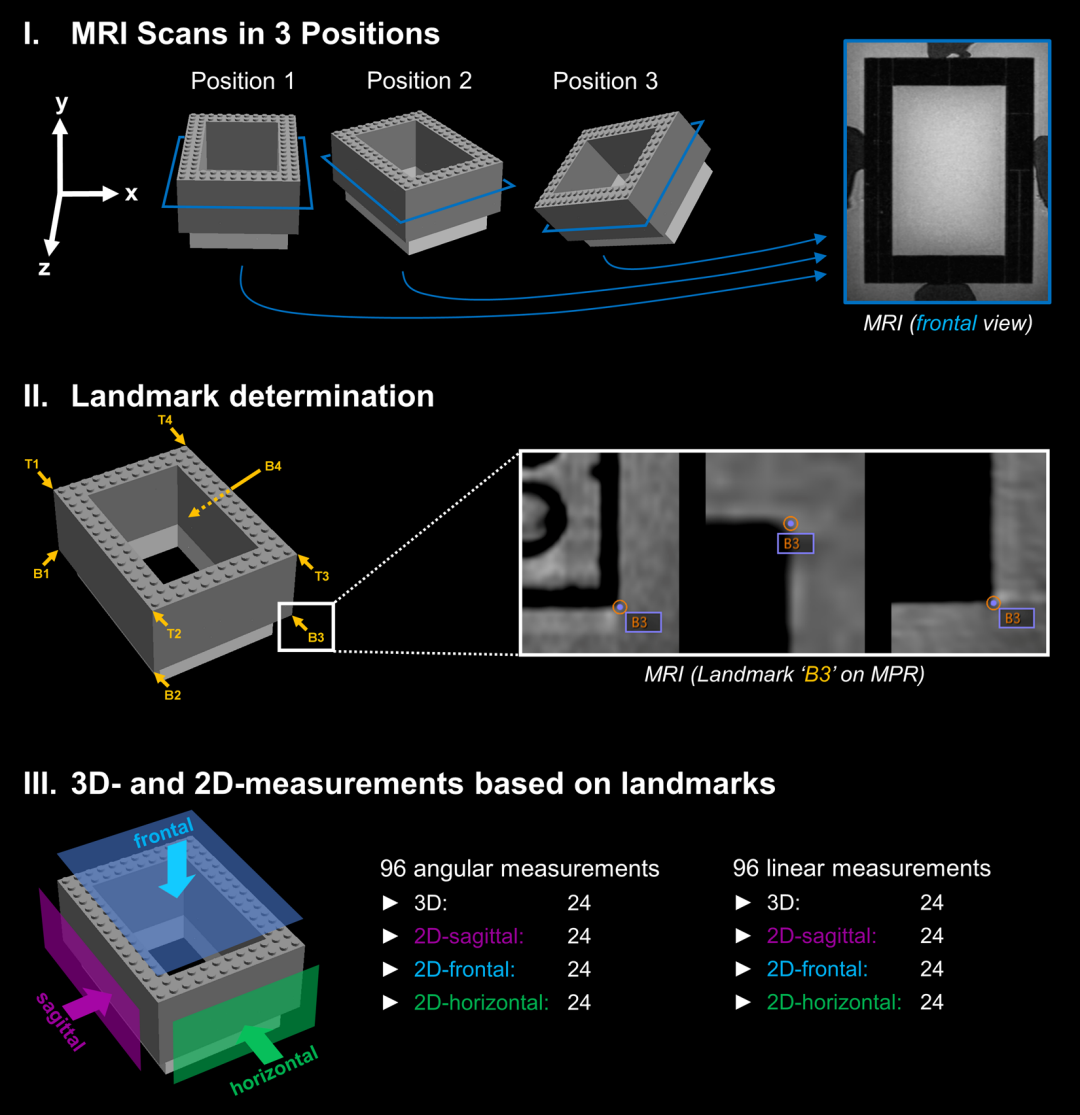
Workflow for analyzing the accuracy of the applied MRI technique. I For accuracy measurements, a cuboid-shaped Lego phantom (127.8 mm × 95.8 mm × 48.0 mm) was scanned on a 3 Tesla MRI system using a high-resolution 3D sequence. The phantom was placed in a plastic box filled with water and contrast agent before it was scanned in three positions (normal alignment, rotated, rotated and lifted). An exemplary MR image in frontal orientation (according to the blue section planes on the phantom graphics) is shown on the right side. II For each phantom position, the 4 vertices on the top (T1–T4) and the 4 vertices on the bottom (B1–B4) were determined as landmarks on multiplanar reconstructions (MPR) using DICOM Imaging Software (Osirix v.7.0.3). III Based on the landmarks’ coordinates, a total of 96 angles and 96 distances were calculated for each phantom position using a customized software tool.

**Figure 2:**
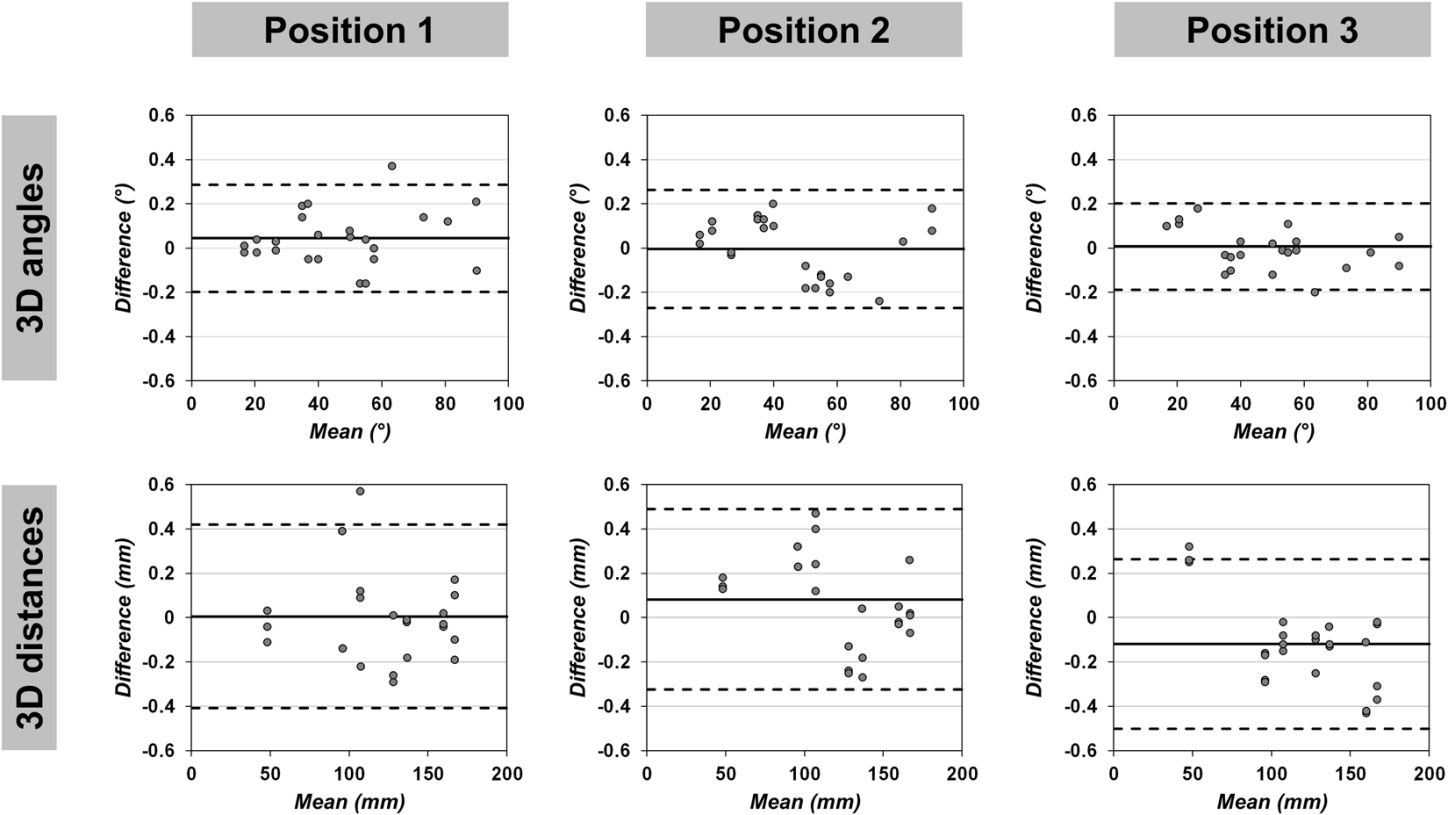
Bland-Altman plots for *in vitro* comparison between true values and 3D MRI measurements. Exemplary Bland-Altman plots for 3D angles and 3D distances in 3 different phantom positions are shown. Solid lines represent the mean of all differences (bias), dashed lines the 95% limits of agreement between the true values and the measurements on MRI.

Bland-Altman analysis revealed excellent agreement and no systematic bias between MRI measurements and true values in all phantom positions (Fig. 1, Tables 1 and 2). Maximum mean differences were 0.04° (95% limits of agreement: −0.20, 0.29) for 3D angles (Position 1), −0.09° (95% limits of agreement: −0.98, 0.76) for 2D angles (horizontal plane orientation in Position 1), −0.12 mm (95% limits of agreement: −0.50, 0.26) for 3D distances (Position 3) and 0.24 mm (95% limits of agreement: −0.13, 0.61) for 2D distances (horizontal plane orientation in Position 2), respectively. Figure 2 shows exemplary Bland-Altman plots for 3D measurements. The high accuracy of MRI based phantom measurements was confirmed by the two one-sided tests (TOST) procedure, which yielded statistical equivalence between MRI and true values in all measurements (all: p < 0.001) at the predefined equivalence margins of ±0.5° and ±0.5 mm, respectively (Tables 1 and 2).

**Table 1:** Accuracy of angular phantom measurements.

Phantom Position	Measurement category	Mean difference (True value − MRI)	95% limits of agreement (True value − MRI)	*p*-value
1	3D angles (°)	0.04	−0.20, 0.29	<0.001
	2D angles sagittal (°)	0.01	−0.36, 0.38	<0.001
	2D angles frontal (°)	0.03	−0.25, 0.31	<0.001
	2D angles horizontal (°)	−0.09	−0.98, 0.76	<0.001
2	3D angles (°)	0.00	−0.27, 0.26	<0.001
	2D angles sagittal (°)	0.04	−0.35, 0.43	<0.001
	2D angles frontal (°)	0.01	−0.26, 0.28	<0.001
	2D angles horizontal (°)	0.03	−0.35, 0.41	<0.001
3	3D angles (°)	0.01	−0.19, 0.20	<0.001
	2D angles sagittal (°)	−0.02	−0.31, 0.27	<0.001
	2D angles frontal (°)	−0.03	−0.20, 0.14	<0.001
	2D angles horizontal (°)	−0.07	−0.47, 0.33	<0.001

All given values refer to 24 different angles per measurement category. p-values < 0.05 indicate equivalence between true values and MRI measurements (two one-sided tests with an equivalence margin of ±0.5°).

**Table 2:** Accuracy of linear phantom measurements.

Phantom Position	Measurement category	Mean difference (True value − MRI)	95% limits of agreement (True value − MRI)	*p*-value
1	3D distances (mm)	0.01	−0.41, 0.42	<0.001
	2D distances sagittal (mm)	−0.06	−0.59, 0.48	<0.001
	2D distances frontal (mm)	−0.01	−0.49, 0.47	<0.001
	2D distances horizontal (mm)	0.11	−0.37, 0.59	<0.001
2	3D distances (mm)	0.08	−0.33, 0.49	<0.001
	2D distances sagittal (mm)	−0.06	−0.48, 0.36	<0.001
	2D distances frontal (mm)	0.03	−0.40, 0.46	<0.001
	2D distances horizontal (mm)	0.24	−0.13, 0.61	<0.001
3	3D distances (mm)	−0.12	−0.50, 0.26	<0.001
	2D distances sagittal (mm)	0.00	−0.40, 0.41	<0.001
	2D distances frontal (mm)	−0.22	−0.46, 0.02	<0.001
	2D distances horizontal (mm)	−0.02	−0.48, 0.43	<0.001

All given values refer to 24 different distances per measurement category. p-values < 0.05 indicate equivalence between true values and MRI measurements (two one-sided tests with an equivalence margin of ±0.5 mm).

**Figure 3:**
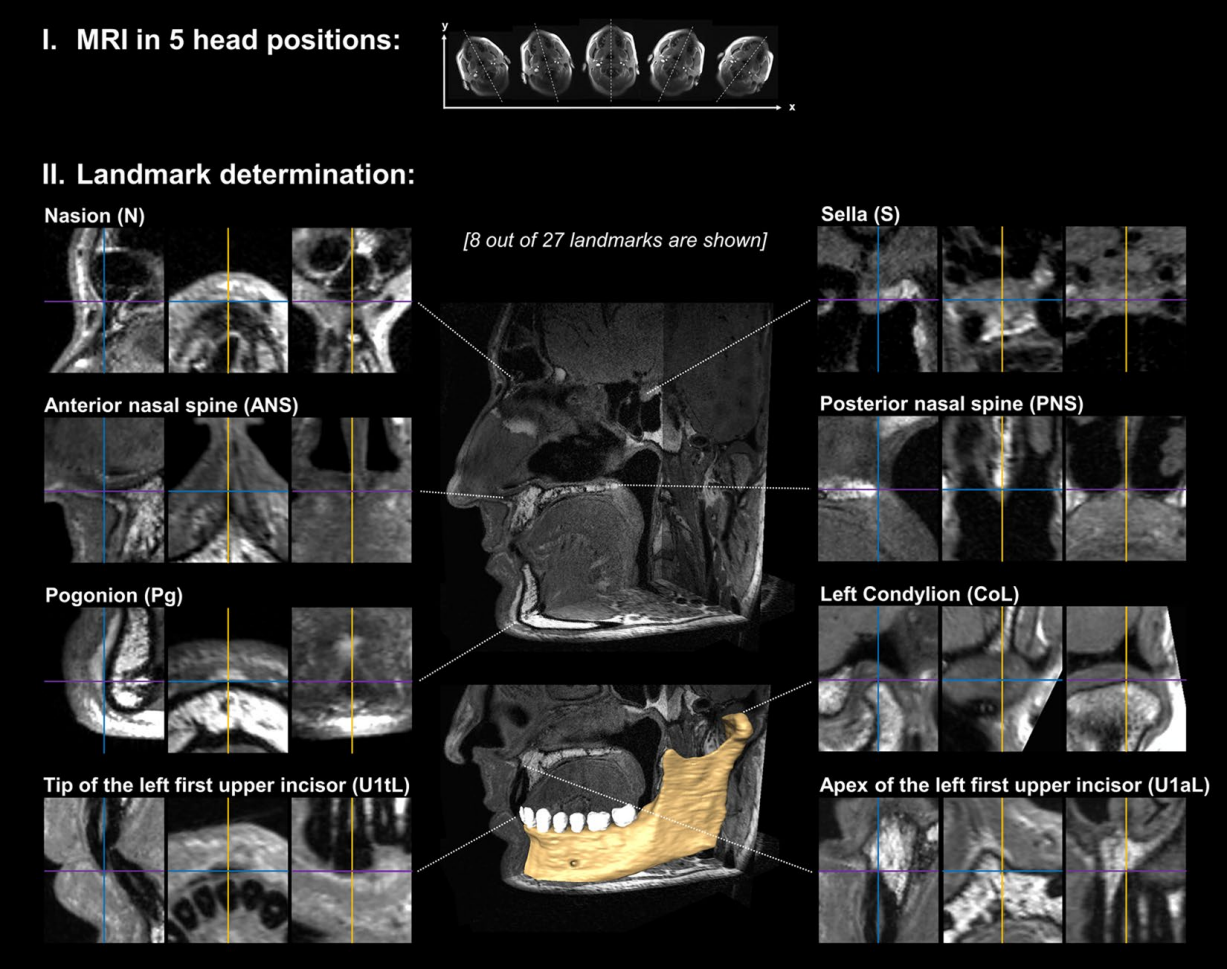
Acquisition of MR images and consecutive landmark determination *in vivo*. I Each volunteer received high-resolution MRI at 3 Tesla in 5 head positions differing in their degree of head rotation. As an example, head positions of volunteer #2 are illustrated by the original axial localizer images. II For each MRI scan, a total of 27 cephalometric landmarks were determined on multiplanar reconstructions (MPR). In this figure, MPR of 8 exemplary landmarks are shown for illustration. The volume segmentation of the left mandible was performed using Amira software (Version 6.4.0, Thermo Fisher Scientific, MA, USA). The full set of cephalometric landmarks is defined and illustrated in Fig. 4.

**Figure 4:**
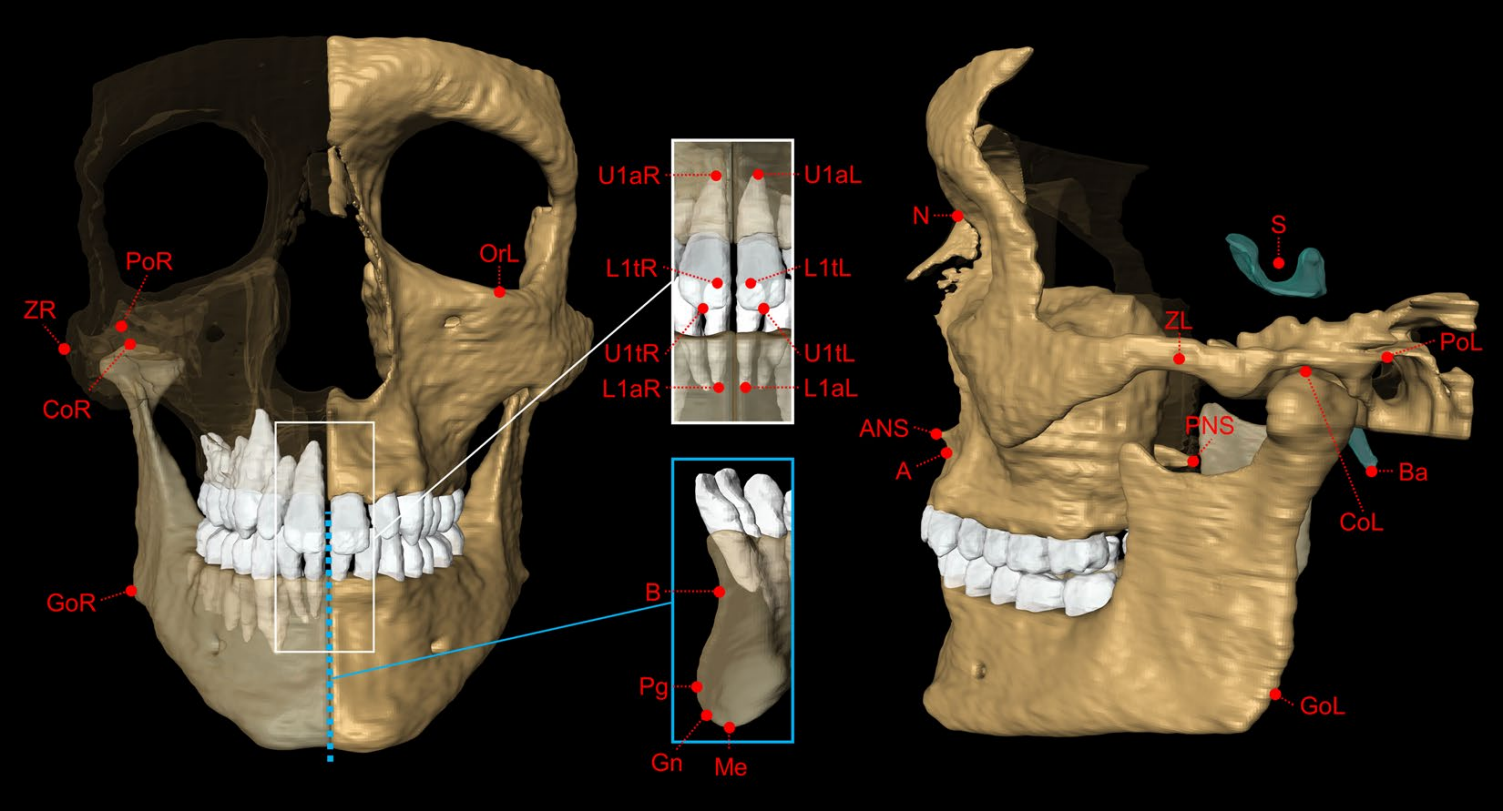
Cephalometric landmarks used in the present study. For each MRI dataset, 27 cephalometric landmarks were determined on multiplanar images. The 3D skull segmentation shown in this figure was generated from 3D MRI of volunteer #2 using Amira software (Version 6.4.0, Thermo Fisher Scientific, MA, USA). Definitions of cephalometric landmarks: N = Nasion (most anterior point of the frontonasal suture); S = Sella (geometric center of the pituitary fossa); Ba = Basion (lowest point on the anterior border of the foramen magnum); CoL/CoR = left/right Condylion (most superior point of Condyle); GoL/GoR = Left/right Gonion (midpoint on the curvature of the angle of the mandible); PoL/PoR = Left/right Porion (Most superior point of the external auditory meatus); OrL = Left Orbitale (most inferior point on the infraorbital margin); ZL/ZR = Left/right Zygion (Most lateral point of the zygomatic arch); ANS = Anterior nasal spine; PNS = Posterior nasal spine; A = Point A (point of maximum midline concavity on the maxilla); B = Point B (point of maximum midline concavity on the mandibular symphysis); Pg = Pogonion (most anterior point of mandibular symphysis); Me = Menton (most inferior point of mandibular symphysis); Gn = Gnathion (Midpoint between Pg and Me); U1tL/U1tR = Tip of the crown of the left/right first upper incisor; U1aL/U1aR = Apex of the left/right first upper incisor; L1tL/L1tR = Tip of the crown of the left/right first lower incisor; L1aL/L1aR = Apex of the left/right first lower incisor; Definitions of planes: N_ANS_PNS = Midsagittal plane defined by the landmarks N, ANS and PNS; PoL_PoR_OrL = Frankfort horizontal plane defined by the landmarks PoL, PoR and OrL.

*In vivo*, MRI-based 3D cephalometric analysis (Figs 3 and 4) showed a high degree of reproducibility across different head positions for each volunteer. There was no statistical difference for repeated measures of angles as well as distances (all: p > 0.05) in repeated measures one-way analysis of variance (ANOVA). For all measurements in all volunteers, average ranges were 0.88° for angles and 0.87 mm for distances. In interindividual comparison, average ranges were at a similar level, with 0.90°/0.89 mm in volunteer #1, 0.90°/0.86 mm in volunteer #2 and 0.83°/0.86 mm in volunteer #3. Largest ranges were 1.69°/1.42 mm (angle N.A.Pg/distances GoR-Me 2D and CR-GN 2D) in volunteer #1, 1.63°/1.68 mm (angle CL.GoL.Me 2D/distance CR-GN 2D) in volunteer #2 and 1.37°/1.43 mm (angle N.S.Ba/distances CL-A 2D and CR-A 2D) in volunteer #3. Mean ranges of MSP oriented 2D measurements were slightly larger in comparison to 3D measurements, with 1.17° (2D) vs. 0.84° (3D), and 1.08 mm (2D) vs. 0.78 mm (3D), respectively. Mean values, standard deviations and ranges for all *in vivo* measurements are shown in Table 3 (angles) and Table 4 (distances).

**Table 3:** Reproducibility of angular *in vivo* measurements.

Measurement	Volunteer #1 MEAN ± SD [RANGE]	Volunteer #2 MEAN ± SD [RANGE]	Volunteer #3 MEAN ± SD [RANGE]
N.S.Ba	128.61 ± 0.45 [1.07]°	126.29 ± 0.26 [0.64]°	125.78 ± 0.49 [1.37]°
S.N.A	83.11 ± 0.25 [0.65]°	83.14 ± 0.37 [0.95]°	84.58 ± 0.21 [0.52]°
S.N.B	81.48 ± 0.15 [0.34]°	80.51 ± 0.30 [0.67]°	81.49 ± 0.27 [0.63]°
S.N.Pg	82.71 ± 0.17 [0.42]°	83.40 ± 0.28 [0.65]°	82.90 ± 0.36 [0.97]°
A.N.B	1.84 ± 0.22 [0.58]°	2.66 ± 0.13 [0.31]°	3.17 ± 0.08 [0.20]°
N.A.Pg	177.83 ± 0.72 [1.69]°	178.61 ± 0.61 [1.57]°	176.43 ± 0.58 [1.27]°
CL.GoL.Me	114.43 ± 0.44 [1.06]°	111.61 ± 0.43 [0.84]°	121.86 ± 0.37 [0.86]°
CL.GoL.Me 2D	118.45 ± 0.19 [0.44]°	111.37 ± 0.61 [1.63]°	122.61 ± 0.44 [1.17]°
CR.GoR.Me	114.18 ± 0.16 [0.43]°	109.14 ± 0.43 [1.15]°	123.71 ± 0.30 [0.80]°
CR.GoR.Me 2D	116.49 ± 0.64 [1.51]°	111.37 ± 0.61 [1.46]°	124.37 ± 0.31 [0.78]°
U1a*mp*-U1t*mp*.L1a*mp*-L1t*mp*	110.82 ± 0.36 [0.88]°	144.81 ± 0.21 [0.54]°	129.71 ± 0.23 [0.56]°
U1a*mp*-U1t*mp*.N-S	114.16 ± 0.30 [0.69]°	97.68 ± 0.53 [1.22]°	107.50 ± 0.34 [0.76]°
U1a*mp*-U1t*mp*.N-A	31.15 ± 0.51 [1.35]°	14.83 ± 0.36 [0.88]°	23.01 ± 0.42 [1.00]°
L1a*mp*-L1t*mp*.Gn-Go*mp*	113.31 ± 0.39 [0.94]°	95.41 ± 0.63 [1.35]°	89.32 ± 0.30 [0.72]°
L1a*mp*-L1t*mp*.B-N	36.46 ± 0.38 [0.92]°	17.99 ± 0.30 [0.71]°	24.19 ± 0.39 [0.99]°
S-N.Go*mp*-Gn	21.73 ± 0.38 [0.77]°	22.06 ± 0.19 [0.45]°	33.37 ± 0.39 [0.90]°
S-N.PNS-ANS	5.79 ± 0.58 [1.27]°	5.06 ± 0.22 [0.53]°	5.64 ± 0.40 [1.04]°
PoL_PoR_OrL.N-Pg	88.46 ± 0.40 [1.10]°	88.63 ± 0.37 [1.03]°	85.09 ± 0.31 [0.70]°
PoL_PoR_OrL.S-Gn	57.42 ± 0.44 [1.03]°	56.24 ± 0.22 [0.55]°	53.89 ± 0.24 [0.51]°
	***p*** = **0.09**	***p*** = **0.31**	***p*** = **0.56**

All angles were calculated from cephalometric landmarks determined on multiplanar reconstructions as shown in Fig. 3. 2D measurements were projected on the midsagittal plane (N_ANS_PNS). All mean, SD and range values refer to 5 MRI scans in different head positions for each study participant. All p-values were generated by repeated measures one-way analysis of variance. p-values > 0.05 indicate no significant differences between the 5 measurement rounds. Definitions of angles: N.S.Ba = Angle between lines N-S and S-Ba (‘cranial base angle’); S.N.A = Angle between lines S-N and N-A; S.N.B = Angle between lines S-N and N-B; S.N.Pg = Angle between lines S-N and N-Pg; A.N.B = angle between lines A-N and N-B; N.A.Pg = Angle between lines N-A and A-Pg; CL.GoL.Me = Angle between lines CL-GoL and GoL-Me (‘left gonial angle’); CR.GoR.Me = Angle between lines CR-GoR and GoR-Me (‘right gonial angle’); U1a*mp*-U1t*mp*.L1a*mp*-L1t*mp* = ‘Interincisal angle’ defined as the angle between maxillary incisors line (the line formed by the midpoint of U1aL/U1aR and the midpoint of U1tL/U1tR) and mandibulary incisors line (the line formed by the midpoint of L1aL/L1aR and the midpoint of L1tL/L1tR); U1a*mp*-U1t*mp*. N-S = angle between maxillary incisors line and line N-S; U1a*mp*-U1t*mp*. N-A = angle between maxillary incisors line and line N-A; L1a*mp*-L1t*mp*.Gn-Go*mp* = angle between mandibular incisors line and the line from Gn to midpoint of GoL and GoR (corresponding to ‘mandibular plane’ in lateral cephalometry); L1a*mp*-L1t*mp*. B-N = angle between mandibular incisors line and the line B-N; S-N.Go*mp*-Gn = angle between line S-N and ‘mandibular plane’; S-N. PNS-ANS = angle between lines S-N and PNS-ANS (corresponding to ‘maxillary plane’ in lateral cephalometry); PoL_PoR_OrL. N-Pg = angle between Frankfort horizontal (FH) plane and line N-Pg (‘facial angle’); PoL_PoR_OrL. S -Gn = angle between FH plane and line S-Gn (‘Y-axis’). Abbreviations: Abbreviations/definitions for cephalometric landmarks and planes are given in the legend of Fig. 4. 2D = two-dimensional; SD = standard deviation.

**Table 4:** Reproducibility of linear *in vivo* measurements.

Measurement	Volunteer #1 MEAN ± SD [RANGE]	Volunteer #2 MEAN ± SD [RANGE]	Volunteer #3 MEAN ± SD [RANGE]
CL-CR	103.20 ± 0.30 [0.65] mm	109.31 ± 0.32 [0.82] mm	106.77 ± 0.22 [0.47] mm
GoL-GoR	95.15 ± 0.50 [1.19] mm	99.64 ± 0.27 [0.66] mm	90.42 ± 0.40 [0.85] mm
ZL-ZR	128.61 ± 0.25 [0.59] mm	136.40 ± 0.24 [0.56] mm	138.86 ± 0.57 [1.18] mm
N-S	72.89 ± 0.14 [0.37] mm	76.46 ± 0.32 [0.62] mm	74.03 ± 0.31 [0.68] mm
S-Ba	43.07 ± 0.25 [0.62] mm	47.85 ± 0.31 [0.80] mm	46.25 ± 0.24 [0.58] mm
PNS-ANS	59.30 ± 0.38 [0.86] mm	61.36 ± 0.35 [0.88] mm	60.89 ± 0.30 [0.75] mm
N-Me	114.52 ± 0.38 [0.95] mm	116.31 ± 0.33 [0.83] mm	123.79 ± 0.38 [0.89] mm
N-ANS	48.45 ± 0.39 [0.97] mm	51.38 ± 0.15 [0.39] mm	51.76 ± 0.33 [0.82] mm
ANS-Me	67.13 ± 0.49 [1.32] mm	65.49 ± 0.19 [0.46] mm	73.93 ± 0.41 [0.98] mm
S-Go*mp*	85.72 ± 0.42 [1.00] mm	84.70 ± 0.31 [0.82] mm	78.28 ± 0.44 [1.13] mm
CL-GoL	64.92 ± 0.54 [1.15] mm	63.45 ± 0.31 [0.83] mm	61.37 ± 0.33 [0.72] mm
CL-GoL 2D	64.88 ± 0.55 [1.15] mm	62.99 ± 0.39 [1.00] mm	60.80 ± 0.34 [0.81] mm
CR-GoR	64.79 ± 0.25 [0.62] mm	62.11 ± 0.45 [0.94] mm	61.42 ± 0.17 [0.38] mm
CR-GoR 2D	64.51 ± 0.20 [0.49] mm	62.07 ± 0.46 [0.98] mm	60.90 ± 0.22 [0.50] mm
GoL-Me	82.66 ± 0.25 [0.60] mm	92.54 ± 0.44 [1.17] mm	90.65 ± 0.41 [1.07] mm
GoL-Me 2D	68.60 ± 0.33 [0.85] mm	77.70 ± 0.22 [0.49] mm	77.76 ± 0.43 [1.07] mm
GoR-Me	81.35 ± 0.19 [0.51] mm	93.74 ± 0.31 [0.79] mm	92.66 ± 0.07 [0.16] mm
GoR-Me 2D	65.01 ± 0.63 [1.42] mm	79.69 ± 0.31 [0.83] mm	81.69 ± 0.44 [1.15] mm
CL-A	101.23 ± 0.23 [0.61] mm	105.10 ± 0.17 [0.42] mm	105.76 ± 0.38 [0.80] mm
CL-A 2D	87.78 ± 0.37 [0.90] mm	88.43 ± 0.47 [1.04] mm	90.57 ± 0.55 [1.43] mm
CR-A	100.12 ± 0.38 [0.82] mm	103.25 ± 0.38 [1.00] mm	108.23 ± 0.26 [0.67] mm
CR-A 2D	85.16 ± 0.54 [1.28] mm	88.91 ± 0.49 [1.27] mm	94.88 ± 0.57 [1.43] mm
CL-Gn	126.03 ± 0.24 [0.56] mm	131.32 ± 0.40 [1.03] mm	136.28 ± 0.32 [0.86] mm
CL-Gn 2D	116.40 ± 0.62 [1.38] mm	117.92 ± 0.50 [1.15] mm	124.54 ± 0.51 [1.28] mm
CR-Gn	124.62 ± 0.33 [0.86] mm	129.59 ± 0.30 [0.82] mm	139.06 ± 0.39 [0.77] mm
CR-Gn 2D	111.93 ± 0.53 [1.42] mm	118.90 ± 0.63 [1.68] mm	129.22 ± 0.39 [0.95] mm
	***p*** = ***0.22***	***p*** = ***0.15***	***p*** = ***0.24***

All distances were calculated from cephalometric landmarks determined on multiplanar reconstructions as shown in Fig. 3. 2D measurements were projected on the midsagittal plane (N_ANS_PNS). All mean, SD and range values refer to 5 MRI scans in different head positions for each study participant. All p-values were generated by repeated measures one-way analysis of variance. p-values > 0.05 indicate no significant differences between the 5 measurement rounds.

Definitions of distances: CL-CR = Distance between points CL and CR (‘bicondylar width’); GoL-GoR = Distance between points GoL and GoR (‘bigonial width’); ZL-ZR = Distance between points ZL and ZR (‘bizygomatic width’); N-S = Distance between points N and S (‘anterior cranial base length’); S-Ba = Distance between points S and Ba (‘posterior cranial base length’); PNS-ANS = Distance between points PNS and ANS (‘palatal length’); N-Me = Distance between points N and Me (‘total anterior facial height’); N-ANS = Distance between points N and ANS (‘upper anterior facial height’); ANS-Me = Distance between points ANS and Me (‘lower anterior facial height’); S-Go*mp* = Distance from point S to midpoint of GoL and GoR (‘posterior facial height’); CL-GoL = Distance between points CL and GoL (‘left mandibular ramus height’); CR-GoR = Distance between points CR and GoR (‘right mandibular ramus height’); GoL-Me = Distance between points GoL and Me (‘left mandibular body length’); GoR-Me = Distance between points GoR and Me (‘right mandibular body length’); CL-A = Distance between points CL and A (‘left midfacial length’); CR-A = Distance between points CR and A (‘right midfacial length’); CL-Gn = Distance between points CL and Gn (‘left mandibular length’); CR-Gn = Distance between points CR and Gn (‘right mandibular length’).

Abbreviations: Abbreviations/definitions for cephalometric landmarks and planes are given in the legend of Fig. 4. 2D = two-dimensional; SD = standard deviation.

## Discussion

In this study, we demonstrated that accurate and precise 3D cephalometric analysis is feasible using non-ionizing, high-resolution MRI. *In vitro* investigations on a phantom showed high concordance between landmark-based MRI measurements and corresponding true values in different phantom positions. *In vivo*, landmark-based 3D cephalometric measurements as applied in clinical routine revealed high levels of reproducibility, independently from the head position of study participants. Our findings indicate that 3D cephalometric analysis could be performed using MRI in the future. This may have a major impact on planning and monitoring of treatment in orthodontic and orthognathic patients, since MRI scans can be performed repeatedly and independently from the extent of malocclusions without radiation exposure to the predominantly young patients.

A key finding of this study was that high measurement accuracy could be demonstrated for the applied MRI technique. Independent from the phantom position, all angular and linear measurements revealed a high concordance with the corresponding true values. Precisely, we found mean differences between true values and MRI ranging from −0.12–0.08 mm for 3D distances, −0.22–0.24 mm for 2D distances, 0.00–0.04° for 3D angles and −0.09–0.04° for 2D angles. This is in line with previous phantom studies analyzing the accuracy of 3D MRI methods designed for craniofacial imaging. Goto *et al*. showed minor differences of 0.2, −0.6 and −0.3 mm between ground truth and 3D MRI measurements using tube phantoms. These differences were only slightly larger compared to identical measurements on CT revealing differences of −0.1, 0.3 and 0.3 mm^19^. Eley *et al*. also demonstrated submillimeter discrepancy between true values and 3D MRI at 3 Tesla for 11 linear measurements performed on a cube phantom, with mean values ranging from 0.02–0.73 mm. In the same study, CT was even slightly less accurate, with ranges from 0.03–0.91 mm compared to ground truth^22^. Overall, our *in vitro* results are in accordance with these studies and confirm high measurement accuracy of our 3D MRI technique which is crucial for the interpretation of subsequent *in vivo* measurements.

MRI offers the unique possibility to repeat *in vivo* examinations for 3D cephalometry as often as required. Given these conditions, we performed 5 MRI examinations in 3 study participants, with different head positions for each scan. Our results revealed a high concordance between repeated measurements in all volunteers. Ranges remained below 2° and 2 mm in all repeated measurements in every study participant. Average ranges were 0.88° for all angles and 0.87 mm for all distances, and if only 3D measurements are included, average ranges were 0.84° and 0.78 mm, respectively. A direct comparison of our results to previous studies is not possible for two reasons: First, comparable MRI studies have not been performed before. Second, for reasons of radiation protection, X-ray based modalities cannot be used to investigate the effect of head positioning on cephalometric measurements *in vivo*. Consequently, cadaver studies using CBCT to analyze 3D cephalometric measurements at varying head orientations are most suitable for comparison. For instance, Ludlow *et al*. performed 4 linear cephalometric measurements on 28 dry skulls in 3 positions (ideal, shifted and rotated), and in comparison to direct skull measurements the absolute value of difference ranged between 0.96 mm and 1.94 mm^23^. Similarly, a CBCT study by Hassan *et al*. assessed 10 linear cephalometric measurements in two different head positions using 8 dry skulls. Their results showed no statistical difference between the two scan positions, with mean absolute differences between CBCT and direct physical measurements ranging from 0.11–0.39 mm for the ideal scan position and 0.10–0.43 mm for the rotated scan position^24^. Overall, results of these cadaver CBCT studies correspond very well with the differences between repeated measurements observed in our *in vivo* MRI study, and thus high *in vivo* reproducibility can be concluded. Importantly, our data for the first time provides *in vivo* evidence that head positioning does not have a significant impact on measurement results in landmark-based 3D cephalometry. Under these favorable conditions, MRI not only provides the possibility to perform longitudinal studies for treatment monitoring but also to specifically examine healthy subjects for the establishment of reference values.

Even though associated with several diagnostic limitations of projection radiography^1–4^, 2D lateral cephalometric analysis will still be used in clinical routine in the future, as various well-established methods and normative data are available^25–29^. Previous *in vivo* studies have already demonstrated that 2D cephalometric analysis can be performed on MRI: In comparison between MRI and lateral cephalometric radiographs (LCR), no clinically relevant discrepancies were observed for 2D analyses including midsagittal^16,30^ as well as bilateral^16^ landmarks. Therefore, lateral 2D measurements defined by a 3-landmark-based midsagittal plane (MSP)^31^ were integrated in the analysis protocol of the present study. This approach revealed a high reliability in repeated 2D measurements with average ranges between minimum and maximum values of 1.17° and 1.08 mm, respectively. From this it can be concluded that high-resolution 3D MR images allow for reproducible lateral cephalometric analysis *in vivo*. As discussed above, our results also demonstrate that the calculated 3D and 2D values do not depend on head orientation. This is a major advantage of 2D MRI measurements in comparison to 2D measurements on cephalometric radiographs, which are susceptible to measurement errors caused by head rotation^2,32,33^.

In view of the results of the present study, it is particularly important to discuss how MRI-based 3D cephalometry could be integrated into clinical practice in the future. Until recently, it was believed that MRI cannot serve as a diagnostic modality for planning of orthodontic therapy or orthognathic surgery^34^. Along with recent technical developments, however, new perspectives have emerged. By combining the latest MRI techniques, we could establish a robust imaging protocol designed for applicability in clinical routine, yielding isotropic images with high resolution and excellent contrast. This was accomplished by using high field MRI (3 Tesla), a 16-channel surface coil and an application-optimized prototype 3D sequence with high spatial resolution. Importantly, the acquisition time of this sequence is only 7:01 minutes and the total examination time lies within approximately 10 minutes including positioning and localizer sequences. Consequently, all *in vivo* scans could be performed time-efficiently with high comfort and no relevant motion artifacts were observed. The acquired high-resolution 3D images allowed a clear depiction of all predefined cephalometric landmarks, resulting in high *in vivo* reproducibility. Analysis of MR images was performed by determining cephalometric landmarks on multiplanar reconstructions (MPR), which means the workflow is identical to MPR-based 3D cephalometric analysis on CT or CBCT images. As with CT/CBCT, the time required for landmark determination depends on the number of predefined landmarks as well as the observer’s training and experience. In the present study, *in vivo* 3D cephalometric analysis included 27 landmarks and was performed by an experienced dentomaxillofacial radiologist within 10–15 minutes per dataset. Altogether, the applied MRI technique enables time-efficient acquisition and analysis of images, thus providing the basis for clinical application of landmark-based 3D cephalometry. As this paper presents a new approach of MRI-based cephalometry with specific technical requirements, availability is limited at this stage. In principle, however, the technique could be established on different MRI systems with similar features for broad clinical use in the future.

Since 3D imaging can substantially improve diagnostic possibilities in orthodontics and orthognathic surgery, many studies have investigated the use of conventional CT and CBCT for 3D measurements of craniofacial structures^35–39^. Recently, particularly CBCT has moved into focus and proven to be an accurate modality for 3D cephalometric analysis^5–7^. However, the use of CBCT for 3D cephalometry is limited because of considerable radiation doses and a substantially increased lifetime attributable cancer risk of young patients^40,41^. As a consequence, reference values for 3D cephalometry are not available until today. In contrast to CBCT, MRI is an imaging modality allowing radiation-free 3D imaging of the craniofacial region which could provide a wide range of new diagnostic options. In view of our results and former *in vitro* studies demonstrating high concordance between measurements on CBCT and MRI^20,21^, these two modalities might deliver equivalent results for 3D cephalometric analysis *in vivo* as well (within clinically acceptable margins).

From a methodological point of view, it is important to stress that artifacts caused by metallic materials (e. g. fixed orthodontic appliances, dental implants or osteosynthesis material) can be a limiting factor of MRI in the craniofacial area^42^. In orthodontics and orthognathic surgery, this could be particularly important for treatment monitoring. To minimize this potential limitation for future patient studies, we used a 3D MSVAT-SPACE sequence which has proven to significantly reduce metal-induced artifacts^15^.

In conclusion, this study demonstrates that high-resolution MRI based on a short examination protocol can be used for 3D cephalometric analysis. The applied MRI technique revealed an excellent accuracy *in vitro* and high levels of reproducibility *in vivo*, independently from the position of investigated object/head. Thus, non-ionizing MRI has the potential to overcome the limitations of X-ray based standard methods, which are the limited diagnostic value of conventional radiographs and the radiation risks associated with CT and CBCT. In absence of radiation exposure, MRI offers the possibility to repeatedly examine patients with varying degrees of orthodontic disorders as well as healthy subjects, which might substantially contribute to provide evidence for the diagnostic and therapeutic efficacy of 3D cephalometry.

## Methods

### Phantom construction

A physical cuboid-shaped phantom with a size of 127.8 mm × 95.8 mm × 48.0 mm was created from Lego bricks (The Lego Group, Billund, Denmark) to evaluate the accuracy of linear and angular measurements based on MRI landmarks (Fig. 1). The true dimensions of the phantom were exactly defined by the size of Lego bricks and additionally confirmed with a digital caliper (PRECISE PS 7215, Burg-Waechter KG, Wetter, Germany).

### Study participants and ethics

For *in vivo* measurements, 3 male participants (27 y, 33 y and 31 y) without relevant craniofacial asymmetries were prospectively enrolled in this study. The study was approved by the institutional ethics committee of the University of Heidelberg (approval number: S-294/2014), written informed consent was obtained from all participants. All methods were performed in accordance with the relevant guidelines and regulations.

### MRI technique and measurements

All phantom and *in vivo* MRI measurements were performed on a 3 Tesla MRI system (MAGNETOM Trio; Siemens Healthcare GmbH, Erlangen, Germany) with a 16-channel multipurpose coil (Variety, Noras MRI products GmbH, Hoechberg, Germany) using a high-resolution T1-weighted 3D MSVAT-SPACE (multiple slab acquisition with view angle tilting gradient based on a sampling perfection with application optimized contrasts using different flip angle evolution) prototype sequence. This MRI sequence allows for 3D high resolution imaging and suppression of susceptibility artifacts at the same time^43^. It was specifically optimized and evaluated for craniofacial MRI, as described elsewhere^15^.

Sequence parameters were: echo time: 5.8 ms, repetition time: 800 ms, bandwidth: 625 Hz/pixel, number of averages: 1, echo train length: 100, field of view: 171 mm × 171 mm, acquisition matrix: 320 × 320, voxel size: 0.53 mm × 0.53 mm × 0.53 mm, number of sections: 256, time of acquisition: 7:01 min.

For accuracy measurements, the Lego phantom was placed in a waterproof plastic box (Lock & Lock, Seoul, South Korea) and fixed in position using silicon impression material (Optosil Comfort, Kulzer GmbH, Hanau, Germany). The plastic box was filled with water and gadoterate (Gd) meglumine contrast (Dotarem®, Guerbet, France) in a ratio of 1:250 to enhance signal from water. Next, the phantom was scanned in 3 positions: 1. “Normal”: Regular alignment in x-, y- and z-direction; 2. “Rotated”: Deviation in x- and z-direction; 3. “Rotated and lifted”: Deviation in x-, y- and z-direction. This setup of phantom scans is illustrated in Fig. 1.

For *in vivo* measurements, each study participant was scanned in centric occlusion in 5 different positions which differed in their degree of head rotation (Fig. 3). Head positions were: 1. Normal (supine position without head rotation), 2. Moderate rotation to the right, 3. Substantial rotation to the right, 4. Moderate rotation to the left and 5. Substantial rotation to the left.

### Analysis of MRI datasets

The acquired MR images were analyzed with the DICOM Imaging Software Osirix v.7.0.3 (Geneva, Switzerland). All predefined phantom and *in vivo* landmarks were identified by AJ (a radiologist with five years’ experience in craniofacial imaging) on MPR images.

For calculation of angular and linear measurements from the identified landmarks, a specific Osirix-plugin was developed by MAS using the software development tool Xcode 9 (Apple Inc., Cupertino, California). The coordinates of landmarks were used to calculate 3D and 2D measurements. For 2D measurements, the observer defined projection planes, each plane based on three landmarks.

Phantom landmarks were the 4 vertices on the top (T1–T4) and the 4 vertices on the bottom (B1–B4) of the cuboid. Each phantom analysis included the calculation of 96 angular (3D: 24, 2D-sagittal: 24, 2D-frontal: 24, 2D-horizontal: 24) and 96 linear measurements (3D: 24, 2D-sagittal: 24, 2D-frontal: 24, 2D-horizontal: 24). For 2D measurements, the coordinate system was oriented according to the sagittal plane (defining landmarks: T1, T2, B2), the horizontal plane (defining landmarks: T1, T4, B4) and the frontal plane (defining landmarks: T1, T2, T4) in all positions (Fig. 1).

For each *in vivo* scan, 27 cephalometric landmarks were determined (Figs 3 and 4) from which 19 angles (3D: 17, 2D: 2) and 26 distances (3D: 18, 2D: 8) were calculated (Tables 3 and 4). The MSP used for 2D lateral cephalometric analysis was defined by the landmarks nasion (N), anterior nasal spine (ANS) and posterior nasal spine (PNS).

### Statistical analysis

Statistical analysis was performed with software (R version 3.4.2; R Foundation for Statistical Computing, Vienna, Austria). For accuracy of phantom measurements, statistical analysis aimed at identifying whether the true values and those measured on MRI were equivalent within a strict predefined equivalence margin [−θ, θ]. For all angular and linear phantom measurements, equivalence testing was carried out by the TOST procedure^44^ with α = 0.05, a 1 − 2α confidence interval and θ = 0.5. Thus, the prespecified acceptable level of difference was ±0.5° and ±0.5 mm, respectively. Null hypothesis of TOST was that the true values and the corresponding MRI measurements were not equivalent. If the 1 − 2α confidence interval was completely contained within the equivalence margin [−θ, θ], the null hypothesis was rejected, and the results were considered equivalent (p-value < 0.05). In addition, the level of agreement between MRI phantom measurements and true values was assessed by Bland-Altman analysis calculating the mean of differences (bias) and the 95% limits of agreement^45^. *In vivo* reproducibility of cephalometric measurements was analyzed by repeated measures one-way ANOVA with Greenhouse-Geisser correction.

## Data Availability

The datasets generated and analyzed during the current study are available from the corresponding author on reasonable requests.
